# PubRunner: A light-weight framework for updating text mining results

**DOI:** 10.12688/f1000research.11389.2

**Published:** 2017-10-13

**Authors:** Kishore R. Anekalla, J.P. Courneya, Nicolas Fiorini, Jake Lever, Michael Muchow, Ben Busby

**Affiliations:** 1 Northwestern University, Chicago, IL, 60611, USA; 2 Health Sciences and Human Services Library, University of Maryland, Baltimore, MD, 21201, USA; 3 National Center for Biotechnology Information, National Institutes of Health, Bethesda, MD, 20894, USA; 4 Canada's Michael Smith Genome Sciences Centre, University of British Columbia, Vancouver, BC, V5Z 4S6, Canada; 5 National Institute of Standards and Technology, Gaithersburg, MD, 20899, USA; 6 National Center for Biotechnology Information, National Library of Medicine, National Institutes of Health, Bethesda, MD, 20894, USA

**Keywords:** PubRunner, PubMed, biomedical text mining, text mining, natural language processing, BioNLP

## Abstract

Biomedical text mining promises to assist biologists in quickly navigating the combined knowledge in their domain. This would allow improved understanding of the complex interactions within biological systems and faster hypothesis generation. New biomedical research articles are published daily and text mining tools are only as good as the corpus from which they work. Many text mining tools are underused because their results are static and do not reflect the constantly expanding knowledge in the field. In order for biomedical text mining to become an indispensable tool used by researchers, this problem must be addressed. To this end, we present PubRunner, a framework for regularly running text mining tools on the latest publications. PubRunner is lightweight, simple to use, and can be integrated with an existing text mining tool. The workflow involves downloading the latest abstracts from PubMed, executing a user-defined tool, pushing the resulting data to a public FTP or Zenodo dataset, and publicizing the location of these results on the public PubRunner website. We illustrate the use of this tool by re-running the commonly used word2vec tool on the latest PubMed abstracts to generate up-to-date word vector representations for the biomedical domain. This shows a proof of concept that we hope will encourage text mining developers to build tools that truly will aid biologists in exploring the latest publications.

## Introduction

The National Library of Medicine’s (NLM) PubMed database contains over 27 million citations and is growing exponentially ( Lu, 2011). Increasingly, text mining tools are being developed to analyze the contents of PubMed and other publicly searchable literature databases. These tools fall into three main categories based on the potential users. The first group of tools is aimed at other text mining researchers to help them solve problems. These tools can assist in parsing (e.g. Stanford CoreNLP - Manning *et al.*), entity recognition (e.g. DNorm - Leaman *et al.*) and other tasks (e.g. Word2Vec - Mikolov *et al.*). The second group of tools is aimed at expert curators to aid their creation of well-maintained biological databases. The third group of tools are aimed directly at biologists and provide automatically generated databases (such as miRTex - Li *et al.*), knowledge discovery capabilities (such as FACTA+ - Tsuruoka *et al.*) and many other uses.

Several challenges face researchers when trying to reuse the biomedical text mining methods and data of other researchers. Firstly, data and particularly code is rarely shared publicly. This is detrimental to the community and makes replicability and reproducibility very challenging. Furthermore, annotation formats and policies vary widely among research groups and specific biological domains. These problems are exacerbated by different ontology usages. These annotation issues limit the interoperability of different research tools and datasets. Finally, the data that is released is often static, as the text mining tool is only executed once and not rerun as new publications are released. This is commonly due to the goal of publishing a paper on the tool after which the tool is forgotten, the graduate student leaves the group and the project is abandoned.

As an example of a tool that would benefit from updated data, the FACTA+ tool ( Tsuruoka *et al.*), which is aimed directly at biologists interested in understanding the associations of a biomedical concept, has not been updated since 2010. Given that it has not been updated, it misses a lot of important data, such as all recent information about Zika outbreaks. Many other tools (e.g. miRTex - Li *et al.*) have been run on a static set of Medline abstracts. Their results are incredibly useful but would prove more valuable if they were updated with the latest publications.

The open science movement has gained momentum in many areas of science. Studies ( McKiernan *et al.*) show that science is stifled by researchers not sharing their data. Efforts such as Zenodo gives researchers an easy-to-use permanent and citable archive in which to store very large datasets. In fact, each dataset can be up to 50GB and is stored on the same robust servers as are used for data from the Large Hadron Collider. The challenge of maintaining up-to-date results requires additional engineering, which often goes beyond a basic research project. Some research is beginning to look at methods to maintain updated analysis on PubMed ( Hakala *et al*.), but a general framework is needed.

To encourage biomedical text mining researchers to widely share their results and code, and keep analyses up-to-date, we present PubRunner. PubRunner is a small framework created during the National Center of Biotechnology Information Hackathon in January 2017. It wraps around a text mining tool and manages regular updates using the latest publications from PubMed. On a regular schedule, it downloads the latest Pubmed files, runs the selected tool(s), and outputs the results to an FTP directory or Zenodo archive. It also updates a public website with information about where the latest results can be located. We feel that this is a small but valuable step to help the text mining community produce robust and widely used tools and will encourage discussion about open data and open source development.

## Methods

**Figure 1.**
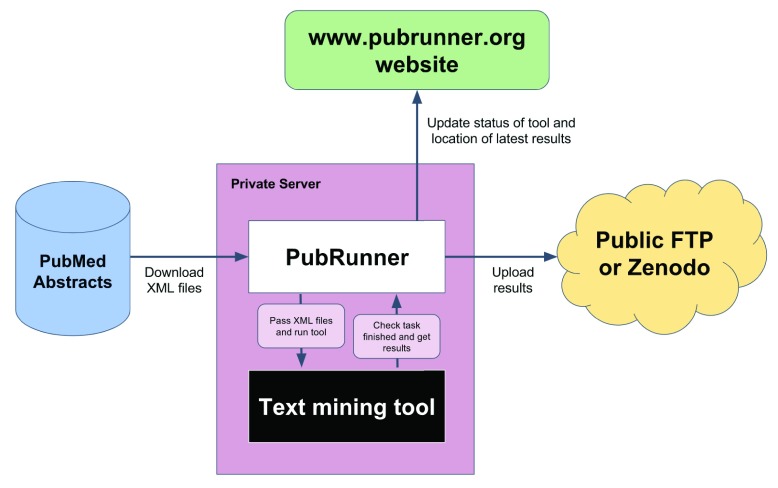
Overview of PubRunner. Overview of PubRunner. PubMed abstract files in XML format are downloaded to the PubRunner framework, processed by the text-mining tools, the output pushed to a public FTP/Zenodo site and an update sent to the central PubRunner website.

PubRunner manages monthly runs of text mining analyses using the latest publications from PubMed without requiring human intervention. The PubRunner framework has several key steps, outlined in Figure 1. First, it queries the PubMed FTP server to identify new XML files and downloads them. It currently downloads the Baseline dataset and then updates with the Daily Updates files ( https://www.nlm.nih.gov/databases/download/pubmed_medline.html). It tracks which files are new and downloads the minimal required set to be up-to-date. Second, it executes the text mining tool(s) on the latest downloaded PubMed files. These tools are then run as Python subprocesses and monitored for exit status. Furthermore, PubRunner uses a timeout parameter to kill processes that exceed a time limit. PubRunner runs on the same private server used for the text mining analysis but moves results to a publicly visible FTP or permanent archiving on Zenodo after the analysis is complete. It requires FTP login information or Zenodo authentication token to be able to upload files.

With this first step, PubRunner requires the tools that it executes to take a set of PubMed XML files. PubRunner does not guarantee any quality measure of the tools that are run. The users of the data generated by a PubRunner run should refer to the publication associated with any tools to understand the quality metrics that the original authors used to gauge the expected performance of the tool.

A central website was developed to track the status of different text mining analyses that are managed by PubRunner. These analyses may be executed on a variety of different researchers’ computers with results hosted on different FTPs. The website lists the tools with information about their latest run and where their code and results can be found. This allows text mining users to more easily find robust and up-to-date analyses on PubMed.

A key design goal of PubRunner is to make installation as straightforward as possible. This is to encourage widespread use of the framework and release of both tool code and results data. Accordingly, a Docker image containing PubRunner has been produced, and installation from the Github code is also very straightforward. Also, each PubRunner component (server, website, and FTP) can be built by using the Docker file available for each in the GitHub repository. Deploying a specific component is thus made easy. Notably, there is not one central PubRunner FTP server. The output of PubRunner can be transferred to a pre-existing FTP server (e.g. an institution’s FTP server) or a new FTP server can be set up using the Docker image. After PubRunner is installed, configuration involves setting the paths to the tools to be run and the login information for the FTP/Zenodo.

PubRunner currently has two dependencies: Python and R. The Docker file manages installation of these tools. The CPU and memory requirements required to run PubRunner depend on the associated text mining tools to be executed. PubRunner does require a reasonable amount of disk space, approximately 185GB, in order to download the full set of PubMed XMLs.

For a text mining tool developer to start using PubRunner, they first register their tool with the central website ( http://www.pubrunner.org). Each tool should accept a set of Medline XML files as input and generate output files in a specific directory. The website gives them instructions on the necessary configuration settings (including an authentication token) so that their PubRunner instance can communicate with the central website. After each scheduled run of PubRunner on their remote server, an update message is sent to the website. This is implemented as an HTTP POST request to a PHP script on the PubRunner website. The request contains a JSON packet of information with an authentication token so that only submissions from authorized users are allowed. The JSON packet includes success status for the tools with URLs to the appropriate data. A potential extension to the website would hide tools that have failed for over three months and send notifications to the maintainers of each failed tool.

## Use case

PubRunner was tested using three test-case text mining tools that were developed specifically for testing the framework and one real-world text mining tool. These tools are also included in the Github repository.

The first of the test-case tools, named CountWords, generated basic word counts for each abstract in a PubMed XML file. It takes as input a list of PubMed XML files, parses the XML for the AbstractText section, splits the text by whitespace and counts the resulting tokens to give a naïve word count. It then outputs the set of word counts along with the corresponding PubMed IDs to a tab-delimited file. In order to test the robustness of the process management, two other tools that would fail were developed. The second tool, simply named Error, consistently failed. The third, named CountWordsError, uses the same code to calculate word counts as the first tool but would fail with a probability of 0.5. PubRunner successfully managed new runs of these test tools using updates from PubMed.

The real-world text mining tool was word2vec ( Mikolov *et al.*). It is a commonly used tool to generate vector representations of individual words. These vector representations are a very commonly used resource in general NLP research and have been used in biomedical text mining ( Mehryary *et al.*). Pyysalo *et al.* created vectors specifically for the biomedical domain in 2013 which are available at. New terms appear frequently and new relations form between biomedical concepts so it is important to update these vectors. We, therefore, built a small pipeline that takes in Pubmed XML files and feeds the raw text of the titles and abstracts from Pubmed citations into the word2vec tool.

At the time of publication, all four tools are deployed using PubRunner on a server hosted by the British Columbia Cancer Agency. PubRunner reruns the tools monthly and updates the results and status posted to the PubRunner website.

## Conclusions and next steps

The PubRunner prototype reduces the additional engineering required for a text mining tool to be run on the latest publications. It will encourage the sharing of tool code and analysis data. At the moment, it can manage text mining runs using the latest Pubmed data. Future versions of the software will add additional corpora sources, such as PubMed Central, allow easier integration of ontologies and other bioinformatics resources and will include the ability to process only a subset of MEDLINE. While this is only the first step towards making biomedical tools easier to use, we hope that it will encourage discussion about how researchers can improve data and code sharing.

## Data and software availability

PubRunner central website: http://www.pubrunner.org

Latest source code for the pipeline is publically available on GitHub: https://github.com/NCBI-Hackathons/PubRunner.

Archived source code as at time of publication: 10.5281/zenodo.892384 ( Lever *et al.*, 2017)

License: MIT

The Docker image is available at https://hub.docker.com/r/ncbihackathons/pubrunner/.
