# Supplementary material for: PubRunner: A light-weight framework for updating text mining results
Source: F1000Res. 2017 Oct 13;6:612. Originally published 2017 May 2. [Version 2] doi: 10.12688/f1000research.11389.2 (PMC5664974; doi:10.12688/f1000research.11389.2)
Supplement: Peer review history

# Peer review: Referee response for version 2

**DOI:** 10.5256/f1000research.13740.r26975

**Published:** 2017-10-31

**Authors:** Julien Gobeill

**Affiliations:** 1 Information Science Department, BiTeM Group, University of Applied Sciences and Arts of Western Switzerland (HES-SO, HEG (Geneva School of Management)), Carouge, Switzerland; 2 Text Mining group, Swiss Institute of Bioinformatics, Geneva, Switzerland

The authors made efforts to improve the article. Regarding the application of biomedical text mining and its potential users, they present admissible and well-founded arguments. The description if the software is now more technically sound. Thus, let's PubRunner find its potential users.

I have read this submission. I believe that I have an appropriate level of expertise to confirm that it is of an acceptable scientific standard.

# Peer review: Referee response for version 1

**DOI:** 10.5256/f1000research.12294.r24597

**Published:** 2017-08-07

**Authors:** Julien Gobeill

**Affiliations:** 1 Information Science Department, BiTeM Group, University of Applied Sciences and Arts of Western Switzerland (HES-SO, HEG (Geneva School of Management)), Carouge, Switzerland; 2 Text Mining group, Swiss Institute of Bioinformatics, Geneva, Switzerland

The authors' assumption is that many text mining tools are underused because their results are static (i.e. not updated with latest publications), and that biological experts could benefit from a centralized platform that would aggregate up-to-date outputs from various text mining tools. In this perspective, they propose and describe a framework: PubRunner.

 The basic idea is simple and naive. Sometimes great things come from simple and naive ideas. I could imagine a centralized platform, where biological experts could explore up-to-date results of different tools, and occasionally download a dataset: it would be quite like Commoncrawl, but for text mining. This idea is quite interesting, as the authors say: "an ecosystem of text mining tools running on the latest publications". Moreover, this could help these developed tools to have a bigger visibility and a longer life.

 Yet, I cannot imagine that this kind of platform could be integrated in a real curation workflow.

 First of all, the authors twice deal with the importance of sharing text mining results: "molecular biologists rely on text mining experts to run these tools on the latest publications and openly share their results". As a text miner, in contact with curators, this is not my life :) In my world, we text miners do not produce results, but tools and/or systems. Curators do not see us as data/results providers: they rather ask for quality-controlled and embedded assist in their curation workflow.

 In a technical perspective, the presented idea has a lot of drawbacks.

 Dealing with data format: what if the tool is not made for working from MEDLINE XML files as input? It seems that each tool would be free to deliver dataset in its own format (xml, json, csv...). How to describe the data content and format for the interested user? Moreover, computing data for all MEDLINE will result in huge dataset. Currently, there is a 260 Mo file just for word counts: would the dataset size still be manageable for more complex output? How to manage systems that are only relevant on a fraction of MEDLINE? (e.g. functional curation)

 Dealing with data quality: how to know the quality of the dataset? This is extremely important for curators.

 Dealing with computation: I don't think it is the job of Pubrunner to download MEDLINE updates (especially all the baseline, which will take days in a good server). "The PubRunner prototype reduces the additional engineering required for a text mining tool to be run on the latest publications": it can be true for text miners that are not used to work in biomedicine. For others, I assume they manage MEDLINE updates by their own. Moreover, I don't think that server managers will fully accept to have a third party platform running, especially on a production server.

 As a conclusion, I think that this work could be the first step to a useful "thing", but it is far from being useful for the moment. The authors conclude that "this will certainly benefit biomedical researchers by allowing easier analysis of the latest publications", and this is not my opinion.

I have read this submission. I believe that I have an appropriate level of expertise to confirm that it is of an acceptable scientific standard, however I have significant reservations, as outlined above.

# Response

**Published:** 2017-09-13

**Authors:** Ben Busby

**Affiliations:** National Center for Biotechnology Information (NCBI), National Institutes of Health, USA

We would like to thank you for taking the time to review this hackathon paper. We have read your comments carefully and have factored them into the changes.

You provided your perspective on the application of biomedical text mining and its potential users. We would suggest that there are broadly three groups of users for text mining tools. Biocurators (those that curate biological databases) are the one that you discussed. They require customised tools that fit their workflows. While some groups do have a text mining expert on staff who is able to provide assistance through the lifetime of the project, other projects may only have an expert for a short-time to start up the initiative. The text mining tools used by these curators provide suggested papers (or more) to the curators. It would seem useful to those groups that their text mining expert could fit the tool in the PubRunner framework so that continued curation suggestions could be provided after the expert has left the project.

However there are two other groups who benefit from text mining tools. There are biologists who use the output of text mining tools directly. These tools can be thought of as fully automated as they are not expertly curated. Understandably, these tools likely provide lower quality information but can be very useful in areas where expert curators cannot be found. Lastly there are text mining tools developed to be used by other text mining researchers (Hakala et al, Mikolov et al). We would suggest that these two groups would benefit greatly from automated updates using the latest publications.

You raise a very valid point with the varied input formats used by different tools. PubRunner, currently expects a tool to accept the Medline XML files. However we have included code that extracts the title and abstract text from Medline XML files. Individual pipelines can make use of this code.

The output data sizes of some text mining tools could be substantial. We have implemented an additional output hosting option with Zenodo. This allows datasets of up to 50GB to be hosted indefinitely. As an example of the ease of this, we added an additional tool (word2vec) that generates files of approximately 5GB.

We agree that some text mining tools need only be run on part of Pubmed. This could be by filtering using MeSH terms, publication dates or other factors. We intend to add this functionality as a future feature and have added it to the paper.

The quality of the output of the data is entirely dependent on the tool. PubRunner isn’t able to make evaluations on the quality of a tool. Users of any data output by a tool would need to refer to the tool’s documentation or associated publication in order to understand the expected quality of output data.

You note that many biomedical text mining groups will already manage the download of MEDLINE baseline and update files. This exemplifies that each group has written their own code for this problem and shows the redundancy between groups. For new researchers, and particularly groups whose main focus is not biomedical text mining, they may not regularly download Pubmed. For a group that already manages the download of PubMed, we intend for a future version of PubRunner to accept the location of an existing download of Pubmed. It will then double check that all files are up-to-date and use that resource (thereby not duplicating downloading efforts).

As a product of a hackathon, we understand that further development is needed in order to make PubRunner production-ready. We have noted this in the conclusion. Many server managers happily run third-party tools on a regular basis and with improved testing and documentation, we feel that a manager could happily run this framework on their own server.

# Peer review: Referee response for version 1

**DOI:** 10.5256/f1000research.12294.r24264

**Published:** 2017-08-07

**Authors:** Fabio Rinaldi

**Affiliations:** 1 Swiss Institute of Bioinformatics, University of Zurich , Zürich, Switzerland

The idea presented in this short paper is clear: PubRunner is a software framework that might help to run text mining pipelines at regular intervals. Besides, Pubrunner is also capable of publishing their results on a reference web site.

 Although PubRunner does probably have some utility, the paper fails to address or even discuss other problems that hinder the reusability of text annotations produced by different text mining systems. On the most basic level, different annotation schemas might be used (e.g. different XML formats, or Json). On an higher levels, different annotation labels might be used for the same entity types, or there might even be partial overlappings between annotation categories used by different systems. Another problems is how to deal with errors and noise introduced by each system. And the list of potential obstacles to integration certainly does not end there.

 Another severe limitation of the paper is that the framework has been tested using only three "toy" scenarios: a word counter, and two error-generating systems. It would have been more interesting to pick at least one real-world text mining system, which perhaps might have prompted the authors to consider other potential problems that the toy systems did not present.

I have read this submission. I believe that I have an appropriate level of expertise to confirm that it is of an acceptable scientific standard, however I have significant reservations, as outlined above.

# Response

**Published:** 2017-09-13

**Authors:** Ben Busby

**Affiliations:** National Center for Biotechnology Information (NCBI), National Institutes of Health, USA

You raise one of the large challenges in biomedical text mining: the different annotation schemes used by different groups. We agree that is one of the limiting factors for the re-use of annotated data. However we argue that text mining tools are not reused because code is not often released, and any results from the tool are left static. We have added a section during the introduction discussing the challenge of reusing others research in biomedical text mining.

We have also added an extra tool, word2vec, which is commonly used to generate word representation vectors for terms. Pyssalo et al (2013) created word vectors using the PubMed corpus, and these have been used by other researchers. However these vectors are now out-of-date. We added the word2vec tool to PubRunner. Furthermore we have added Zenodo as an option for hosting the resulting data. This allows the large files generated by word2vec to be hosted indefinitely.

# Peer review: Referee response for version 1

**DOI:** 10.5256/f1000research.12294.r22433

**Published:** 2017-05-26

**Authors:** Jin-Dong Kim

**Affiliations:** 1 Database Center for Life Science (DBCLS), Research Organization of Information and Systems, Tokyo, Japan

Overall, I like the idea of PubRunner. However, as a potential user of the tool, I have three problems described below.

1. While the motivation of the work is clear, what was not clear to me was the benefit of using a specialized tool like PubRunner instead of utilizing a more general scheduling tool like cron. I guess the authors know the benefits, but as it was not clear in the manuscript, I suggest the authors to explain it more clearly in the manuscript to better motivate potential users actually to choose to use PubRunner over alternatives.
2. In the manuscript, the protocol between an instance of PubRunner and the "central website" is not clearly described. It is even completely missing in Figure 1. I think designing such a protocol is not a trivial work, and it has be to clearly explained in the manuscript.
3. For the PubRunner to be more useful, I also think the method of publishing the results has to be more generalized. In fact, I have a big question about requiring the results to be uploaded to a FTP server. Can't it be a HTTP server? If PubRunner allows its user to choose the way of publishing the text mining results, it will become much more useful. This problem is actually related to the problem 1, e.g., if I choose to utilize cron, I can freely choose how to publish the result of my text mining pipeline.

I have read this submission. I believe that I have an appropriate level of expertise to confirm that it is of an acceptable scientific standard, however I have significant reservations, as outlined above.

# Response

**Published:** 2017-09-13

**Authors:** Ben Busby

**Affiliations:** National Center for Biotechnology Information (NCBI), National Institutes of Health, USA

The reason to use the PubRunner framework over a simple CRON job is that PubRunner manages the download of files and upload of results files to a publicly available location. While many research groups have previously written code to manage the download of the PubMed baseline, there is incredible redundancy across these groups. It is worthwhile to create one implementation that can be used by existing groups or new groups as they start work in the biomedical text mining group.

We have added further details of the communication protocol between the PubRunner tool and the website ( http://www.pubrunner.org) which is used to update the current status and information about data location.

You had also questioned the ability to only upload to FTP sites. Most HTTP websites will have an FTP access point which could be used by PubRunner. Furthermore, we have added functionality to upload to Zenodo which is a permanent data repository. It allows datasets up to 50GB, is hosted on the same infrastructure as the Large Hadron Collider and provides Digital Object Identifiers (DOIs) to allow PubRunner produced data to be easily citable.
